# Alcohol Consumption and Risk of Dementia and Cognitive Decline Among Older Adults With or Without Mild Cognitive Impairment

**DOI:** 10.1001/jamanetworkopen.2019.10319

**Published:** 2019-09-27

**Authors:** Manja Koch, Annette L. Fitzpatrick, Stephen R. Rapp, Richard L. Nahin, Jeff D. Williamson, Oscar L. Lopez, Steven T. DeKosky, Lewis H. Kuller, Rachel H. Mackey, Kenneth J. Mukamal, Majken K. Jensen, Kaycee M. Sink

**Affiliations:** 1 Department of Nutrition, Harvard T.H. Chan School of Public Health, Boston, Massachusetts; 2 Department of Family Medicine, University of Washington, Seattle; 3 Department of Epidemiology, University of Washington, Seattle; 4 Department of Global Health, University of Washington, Seattle; 5 Department of Psychiatry and Behavioral Medicine, Wake Forest School of Medicine, Winston-Salem, North Carolina; 6 National Center for Complementary and Integrative Health, National Institutes of Health, Bethesda, Maryland; 7 Department of Internal Medicine, Wake Forest School of Medicine, Winston-Salem, North Carolina; 8 Department of Neurology, School of Medicine, University of Pittsburgh, Pittsburgh, Pennsylvania; 9 Department of Neurology, University of Florida, Gainesville; 10 Department of Epidemiology, Graduate School of Public Health, University of Pittsburgh, Pittsburgh, Pennsylvania; 11 Department of Medicine, Beth Israel Deaconess Medical Center, Boston, Massachusetts; 12 Department of Epidemiology, Harvard T.H. Chan School of Public Health, Boston, Massachusetts; 13 Genentech, South San Francisco, California

## Abstract

**Importance:**

Substantial heterogeneity and uncertainty exist in the observed associations between alcohol consumption and dementia.

**Objective:**

To assess the association between alcohol consumption and dementia and the roles of mild cognitive impairment (MCI) and apolipoprotein E ε4 (*APOE E4*) genotype in modifying this association.

**Design, Setting, and Participants:**

This cohort study used data from the Ginkgo Evaluation of Memory Study, conducted from 2000 to 2008 among US community-dwelling participants. This study analyzed 3021 participants aged 72 years and older who were free of dementia. Data analysis was performed from 2017 to 2018.

**Exposures:**

Self-reported alcohol consumption, drinking frequency, and quantity.

**Main Outcomes and Measures:**

Using multivariable proportional hazards regression and linear mixed models, the risk of dementia and the rate of change over time in the Modified Mini-Mental State Examination were estimated.

**Results:**

Among 3021 participants, the median (interquartile range) age was 78 (76-80) years; 1395 (46.2%) were female. During a median (interquartile range) follow-up of 6.0 (4.9-6.5) years, 512 cases of dementia occurred. For 7.1 to 14.0 drinks per week compared with less than 1.0 drink per week, the hazard ratios for dementia were 0.63 (95% CI, 0.38-1.06) among 2548 participants without MCI and 0.93 (95% CI, 0.47-1.84) among 473 participants with MCI. Among participants with MCI, the hazard ratio for dementia was 1.72 (95% CI, 0.87-3.40) for more than 14.0 drinks per week compared with less than 1.0 drink per week. The association of alcohol intake with dementia differed for participants with and without baseline MCI (*P* for interaction = .03). Among participants without MCI, daily low-quantity drinking was associated with lower dementia risk than infrequent higher-quantity drinking (hazard ratio, 0.45; 95% CI, 0.23-0.89; *P* = .02). Findings were consistent when stratified by sex, age, and *APOE E4* genotype. Compared with drinking less than 1.0 drink per week, complete abstention (in participants without MCI) and the consumption of more than 14.0 drinks per week (in participants with MCI) were associated with lower Modified Mini-Mental State Examination scores (mean difference at follow-up compared with baseline, −0.46 point [95% CI, −0.87 to −0.04 point] and −3.51 points [95% CI, −5.75 to −1.27 points], respectively).

**Conclusions and Relevance:**

In this study, complete abstention and consuming more than 14.0 drinks per week (compared with drinking <1.0 drink per week) were associated with lower cognitive scores among participants aged 72 years and older. Particular caution is needed among individuals with MCI who continue to drink alcohol.

## Key Points

**Question:**

Is alcohol consumption associated with the risk of dementia and cognitive decline in older adults with or without mild cognitive impairment?

**Findings:**

In this cohort study of 3021 participants aged 72 years and older, alcohol intake within recommended limits was not significantly associated with a lower risk of dementia among participants with or without mild cognitive impairment at baseline. Among participants without mild cognitive impairment, daily low-quantity drinking was associated with lower dementia risk compared with infrequent higher-quantity drinking.

**Meaning:**

These findings suggest that physicians caring for older adults need to carefully assess the full dimensions of drinking behavior and cognition when providing guidance to patients about their alcohol consumption.

## Teaser

This cohort study examines the association between alcohol intake and risk of dementia among older adults with or without mild cognitive impairment.

## Introduction

Given the rapidly growing burden of Alzheimer disease (AD) and other dementias, including 50 million people currently living with dementia and 82 million expected by 2030,^1^ the identification of factors that prevent or delay the onset of dementia remains of paramount concern. Several epidemiologic studies have shown that consumers of moderate amounts of alcohol have a lower dementia risk compared with nondrinkers, similar to the corresponding association with lower cardiovascular disease risk.^2^ However, substantial heterogeneity in the observed associations exists,^3^ and several aspects remain unclear.

First, we know little about the independent associations of quantity and frequency of alcohol consumption with dementia risk. Previous studies^4^ have focused on the combined measure of total alcohol consumption, although quantity and frequency differ substantially in their associations with other chronic disease outcomes. Similar to the adverse associations of long-term excessive drinking with dementia risk,^5^ an episodic drinking pattern with higher amounts of alcohol consumed per drinking day might be expected to particularly increase dementia risk. Second, some studies^6,7^ suggest that the associations of alcohol consumption with dementia risk may vary by the absence or presence of the apolipoprotein E ε4 (*APOE E4*) allele, but reports are conflicting. Third, it remains uncertain whether the association of alcohol consumption with dementia may differ among the growing population of older adults living with mild cognitive impairment (MCI).^8^ According to the National Institute on Alcohol Abuse and Alcoholism,^9^ adults aged 65 years and older who are healthy should not have more than 3 drinks on a given day or 7 drinks in a week, but, to our knowledge, no guidelines for patients with MCI exist. Some studies^10,11^ link moderate alcohol consumption to less cognitive decline and lower dementia risk, whereas 1 study^12^ suggested that even consumption of light-to-moderate amounts of alcohol, compared with nondrinking, may be associated with an increased risk of AD in adults with MCI, raising concern about its safety in this population.

To address these issues, we examined the association of self-reported alcohol consumption with the incidence of dementia and cognitive decline in a large, US community-dwelling population of older adults who underwent systematic cognitive function testing and determination of dementia and the roles of MCI and *APOE E4* genotype in modifying this association.

## Methods

### Study Population and Design

We conducted a prospective cohort study of 3069 participants enrolled in the Ginkgo Evaluation of Memory Study (GEMS),^13^ which was a randomized, double-blind, placebo-controlled clinical trial (NCT00010803) conducted from 2000 to 2008 evaluating the association between *Ginkgo biloba* and the prevention of dementia in older adults. The trial identified no overall association between *G biloba *and dementia,^14^ but the study provides an extraordinary resource for investigating alcohol intake in association with subsequent risk of dementia because of its robust dedication of resources to detecting subtle incident neurocognitive changes. Participants were recruited from 4 academic medical centers in the United States. Study participants were aged 72 years or older at baseline and underwent cognitive and functional assessments for incident dementia during a median (interquartile range) follow-up of 6.0 (4.9-6.5) years. Entry criteria for GEMS excluded individuals who would be unable, in the judgment of study staff, to complete the trial, including those with a known history of excessive alcohol use.

The initial trial was conducted in compliance with the Declaration of Helsinki. The institutional review boards of all 4 academic medical centers approved this study, and participants provided written informed consent. Data analysis was performed from July 2017 to June 2018. This study follows the Strengthening the Reporting of Observational Studies in Epidemiology (STROBE) reporting guideline.

### Alcohol Consumption

At the baseline visit, participants reported their frequency of beer, wine, and liquor consumption in days per week and their usual number of 12-oz cans or bottles of beer, 6-oz glasses of wine, and shots of liquor consumed on each occasion. We categorized participants according to their alcohol consumption as follows: none, less than 1.0 drink per week, 1.0 to 7.0 drinks per week, 7.1 to 14.0 drinks per week, and more than 14.0 drinks per week. In the absence of information on previous drinking, participants consuming less than 1.0 drink per week were set as the reference group, as recommended by Shaper et al^15^ and others.^16^

### Cognitive Assessments and Determination of Incident Dementia

In brief, each participant underwent a comprehensive neuropsychological battery of 10 tests at study screening. Between 2000 and 2008, the Modified Mini-Mental State Examination (3MSE) and the Clinical Dementia Rating scale were administered every 6 months through the end of follow-up, death, or dementia diagnosis, whichever occurred first. Through August 2004, the Alzheimer’s Disease Assessment Scale cognitive portion (ADAS-Cog) was administered every 6 months and then annually thereafter. Results of the 3MSE, Clinical Dementia Rating, or ADAS-Cog triggered the comprehensive battery if performance met predefined thresholds.^13^ From 2004 until the study completion in June 2008, all participants underwent the comprehensive neuropsychological battery annually.^17^

By use of an algorithm of neuropsychological test scores, site neurologists examined participants with potential cognitive dysfunction, who then underwent cerebral magnetic resonance imaging. Potential cases were then referred to an expert panel of clinicians who reviewed all the clinical and cognitive data and used a validated adjudication process to determine the presence of all-cause dementia (*Diagnostic and Statistical Manual of Mental Disorders IV* criteria^14^) or MCI at each visit according to the consensus guidelines from the International Working Group on Cognitive Impairment.^18^ For the purposes of diagnosis, MCI was defined as scoring less than or equal to the 10th percentile for age and education level on 2 or more of the 10 neuropsychological tests using the Cardiovascular Health Study population as a reference^19^ while also having a Clinical Dementia Rating global score of 0.5. National Institute of Neurological and Communicative Disorders and Stroke–Alzheimer’s Disease and Related Disorders Association criteria^20^ were used to classify AD specifically.

### Other Covariates

Trained technicians assessed age, sex, years of education, and race/ethnicity in interviews at the screening visit. Assessments included *APOE* genotyping from DNA isolated from blood samples obtained at baseline. Furthermore, trained technicians measured body weight, height, and blood pressure and assessed smoking status and history of heart disease and diabetes in interviews. Participants brought prescription medicine and over-the-counter drugs to the clinic visit. The Center for Epidemiologic Studies Depression Scale was used to assess depressive symptoms. Treatment assignment (placebo or *G biloba*) was determined by permuted-block design by study site, and allocation has been unblinded since study closure. During follow-up, medical records of fatal events were collected to record deaths from any cause.^13^

We used the information on the frequency of engagement in playing cards and visiting with others to estimate the degree of social interaction. Participants underwent phlebotomy at the screening visit, and frozen plasma samples were shipped on dry ice to the Nutritional Biomarker Laboratory at the Harvard T.H. Chan School of Public Health, where high-density lipoprotein cholesterol levels were measured using an enzymatic assay after precipitation of apoB lipoproteins (Thermo Fisher Scientific) and apoA-1 levels were measured using a sandwich enzyme-linked immunosorbent assay (Academy Biomedical, Company Inc).^21^

### Statistical Analysis

To validate alcohol consumption at the group level, we assessed the sex-adjusted correlations of high-density lipoprotein cholesterol (1519 participants) and apoA-1 (1517 participants) levels with the number of drinks.^22^ We used Cox proportional hazards models to assess the risk of all-cause dementia, as well as AD specifically (censoring non-AD cases at the time of dementia diagnosis), according to alcohol consumption with age as the underlying time axis. We initially adjusted for sex, race/ethnicity (white or nonwhite), and clinic site. Subsequent analyses also controlled for education (years), social activity (never or <1 time per month, 1-3 times per month, or ≥1 times per week), smoking status (never, former, current, or missing), body mass index (calculated as weight in kilograms divided by height in meters squared; underweight, <20; normal, 20-24.9; overweight, 25-29.9; obese, ≥30; or missing), use of lipid-lowering medication (former, current, or never), history of cardiovascular disease, presence of diabetes, Center for Epidemiologic Studies Depression Scale score (continuous), treatment assignment (placebo or *G biloba*), and *APOE* genotype (*E22*,* E23*,* E24*,* E33*,* E34*,* E44*, or missing). To test whether observed associations varied by age, sex, *APOE E4* carrier status, or MCI at baseline, we included their separate interaction terms. To test linear and quadratic trends, we used alcohol consumption as a simple continuous variable, winsorizing all values greater than the 97.5th percentile at the 97.5th percentile. We tested the proportional hazards assumption on the basis of Schoenfeld residuals. We also assessed dementia risk according to alcohol consumption using Fine and Gray^23^ proportional subhazard regression to determine the sensitivity of our results to the competing risk of death. To illustrate nonlinear associations further, we plotted the number of drinks and risk for dementia using proportional hazards generalized additive models (3 *df*). We determined whether alcoholic beverage types differed in their association with risk of all-cause dementia and AD specifically (eAppendix 1 and eAppendix 2 in the Supplement).

We assessed adjusted mean differences in global cognitive function as measured by 3MSE and ADAS-Cog scores at follow-up compared with baseline according to alcohol consumption by using multivariable adjusted linear mixed models with a random intercept and random slope for each individual. We excluded the first administration of tests from the analysis to account for practice effects. Thus, the 6-month visit scores were used as the baseline. We then compared adjusted mean values at all subsequent visits, controlling for baseline. To test whether observed associations varied by *APOE E4* carrier status or MCI at baseline, we included their separate interaction terms. All statistical tests were 2-tailed, and *P* < .05 was considered statistically significant. We performed all analyses using Stata statistical software version 12.1 (StataCorp).

## Results

### Baseline Characteristics

Of the initial 3069 participants (2587 without MCI and 482 with MCI), we excluded 48 with insufficient data to calculate usual alcohol intake, leaving 3021 participants in the final analysis. Among the 3021 participants, the median (interquartile range) age was 78 (76-80) years; 1395 (46.2%) were female. At baseline, 58% of participants reported consuming alcohol. Higher reported alcohol consumption tended to be associated with a higher likelihood of being male and a former or current smoker (Table 1). The prevalence of diabetes and MCI at baseline were highest among nondrinkers, but approximately 45% of participants with MCI reported current alcohol consumption. Higher reported alcohol consumption was associated with higher levels of high-density lipoprotein cholesterol (sex-adjusted correlation coefficient, *r* = 0.18; *P* < .001) and higher levels of apoA-1 (*r* = 0.20; *P* < .001) to expected degrees.^24^ During follow-up, 57 participants (12%) with MCI at baseline and 320 participants (13%) without MCI at baseline died. A total of 1.1% of participants were lost to follow-up per year because of deaths and other losses to follow-up (the overall loss to follow-up rate for GEMS was 6.3%).^14^

**Table 1.** Characteristics of 3021 Participants of the Ginkgo Evaluation of Memory Study According to Usual Alcohol Consumption

Characteristic	Participants, No. (%)				
	Nondrinkers (n = 1286)	0.1-0.9 Drinks/wk (n = 466)	1.0-7.0 Drinks/wk (n = 689)	7.1-14.0 Drinks/wk (n = 286)	>14.0 Drinks/wk (n = 294)
Weekly drinks, median (IQR), No.	0 (0-0)	0.2 (0.06-0.46)	2.0 (1.2-4.0)	8.2 (7.5-14.0)	16.0 (14.7-21.5)
Wine	0 (0-0)	0.2 (0.02-0.23)	1.0 (0.2-1.0)	7.0 (0.2-7.0)	2.0 (0.2-14.0)
Beer	0 (0-0)	0 (0-0.02)	0 (0-1.0)	0.2 (0-1.0)	0.2 (0-2.0)
Liquor	0 (0-0)	0 (0-0.08)	0.2 (0-1.0)	0.7 (0-7.0)	14.0 (0.5-14.0)
Age, median (IQR), y	78 (76-81)	78 (76-80)	78 (76-80)	78 (76-81)	77 (76-80)
Female	716 (56)	239 (51)	288 (42)	92 (32)	60 (20)
White	1199 (93)	446 (96)	666 (97)	281 (98)	292 (99)
Education, median (IQR), y	13 (12-16)	14 (12-17)	15 (12-17)	16 (13-17)	16 (13-17)
Engagement in social activity					
Never or <1 time/mo	85 (7)	26 (6)	28 (4)	15 (5)	13 (4)
1-3 times/mo	430 (33)	160 (34)	235 (34)	105 (37)	111 (38)
≥1 times/wk	771 (60)	280 (60)	426 (62)	166 (58)	170 (58)
Body mass index^a^					
Underweight	36 (3)	6 (1)	14 (2)	2 (1)	5 (2)
Normal weight	369 (29)	142 (31)	219 (32)	104 (36)	77 (26)
Overweight	566 (44)	185 (40)	340 (49)	139 (49)	158 (54)
Obese	308 (24)	132 (28)	116 (17)	40 (14)	51 (18)
Smoking status^b^					
Never	669 (53)	200 (44)	205 (30)	81 (29)	53 (18)
Former	551 (44)	231 (51)	438 (65)	191 (68)	214 (74)
Current	42 (3)	23 (5)	36 (5)	10 (4)	24 (8)
History					
Cardiovascular disease	425 (33)	154 (33)	220 (32)	98 (34)	94 (32)
Diabetes	152 (12)	43 (9)	48 (7)	17 (6)	16 (5)
Diastolic blood pressure, median (IQR), mm Hg	69 (61-75)	69 (62-74)	69 (61-76)	69 (62-75)	70 (62-77)
Lipid-lowering medication use	375 (29)	139 (30)	180 (26)	72 (25)	76 (26)
Center for Epidemiologic Studies Depression Scale score, median (IQR)	3 (1-6)	3 (1-5)	3 (1-5)	3 (1-5)	3 (1-5)
Death during follow-up	167 (13)	51 (11)	89 (13)	30 (10)	40 (14)
Mild cognitive impairment	260 (20)	60 (13)	89 (13)	36 (13)	28 (10)
Modified Mini-Mental State Examination score, median (IQR)	93 (89-96)	95 (92-98)	95 (91-97)	95 (91-98)	95 (92-97)
Alzheimer’s Disease Assessment Scale cognitive score, median (IQR)	6 (5-8)	6 (4-8)	6 (5-8)	6 (5-8)	6 (5-8)
*APOE E4* allele carrier^c^	243 (24)	97 (26)	141 (26)	47 (21)	43 (18)
Dementia	254 (20)	74 (16)	103 (15)	38 (13)	43 (15)
Alzheimer	169 (13)	46 (10)	76 (11)	29 (10)	28 (10)
Vascular	7 (1)	7 (2)	6 (1)	1 (<1)	2 (1)
Mixed	67 (5)	17 (4)	18 (3)	6 (2)	11 (4)
Other	11 (1)	4 (1)	3 (<1)	2 (1)	2 (1)

Abbreviation: IQR, interquartile range.

^a^ Sample size was 3009. Body mass index is calculated as weight in kilograms divided by height in meters squared (underweight, <20; normal, 20-24.9; overweight, 25-29.9; obese, ≥30).

^b^ Sample size was 2968.

^c^ Sample size was 2416.

### Alcohol Consumption and Incident Dementia

During a median (interquartile range) follow-up of 6.0 (4.9-6.5) years, 512 cases of dementia occurred, including 348 cases of AD. The presence of MCI at baseline modified the association of alcohol consumption and dementia risk (*P* for interaction = .03). The Figure shows smoothed functions for dementia risk by alcohol consumption and baseline cognitive status. Among participants without MCI at baseline, no category of consumption was significantly associated with higher risk compared with consumption of less than 1 drink per week. The hazard ratio (HR) for dementia for nondrinking compared with light drinking was 1.17 (95% CI, 0.84-1.62; *P* for quadratic trend = .07) (Table 2).

**Figure.**
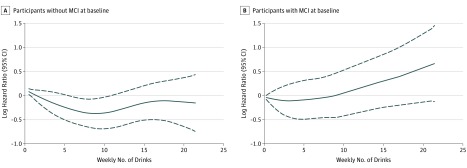
Risk of Dementia in Participants With and Without Mild Cognitive Impairment (MCI) at Baseline by Self-reported Weekly Number of Drinks Plots show log hazard ratios (solid lines) and 95% confidence intervals (dashed lines) for risk of dementia by self-reported weekly number of drinks among 2548 participants without MCI at baseline (A) and 473 participants with MCI at baseline (B). Log hazard ratios were obtained from a generalized additive proportional hazards model with 3 *df* adjusted for age, sex, race/ethnicity, clinic site, educational level, social activity, smoking status, body mass index, lipid-lowering medication use, history of cardiovascular disease, diabetes, Center for Epidemiologic Studies Depression Scale score, treatment group assignment, and *APOE* genotype.

**Table 2.** Adjusted HRs of Dementia According to Usual Alcohol Consumption Among 3021 Participants of the Ginkgo Evaluation of Memory Study^a^

Characteristic	Weekly No. of Drinks					*P* Value for Trend	
	Nondrinkers	0.1-0.9	1.0-7.0	7.1-14.0	>14.0	Linear	Quadratic
No cognitive impairment at baseline (n = 2548)							
Participants, No.							
With dementia	151	50	68	22	26		
Without dementia	875	356	532	228	240		
HR (95% CI)							
Basic model	1.14 (0.82-1.57)	1 [Reference]	0.94 (0.65-1.35)	0.67 (0.40-1.11)	0.87 (0.54-1.42)	.10	.17
Full model	1.17 (0.84-1.62)	1 [Reference]	0.88 (0.61-1.28)	0.63 (0.38-1.06)	0.91 (0.56-1.48)	.10	.07
Mild cognitive impairment at baseline (n = 473)							
Participants, No.							
With dementia	103	24	35	16	17		
Without dementia	157	36	54	20	11		
HR (95% CI)							
Basic model	1.02 (0.65-1.60)	1 [Reference]	0.97 (0.57-1.66)	1.13 (0.58-2.17)	1.63 (0.86-3.11)	.11	.81
Full model	0.98 (0.62-1.56)	1 [Reference]	0.90 (0.52-1.57)	0.93 (0.47-1.84)	1.72 (0.87-3.40)	.13	.40

Abbreviation: HR, hazard ratio.

^a^ HRs were obtained from Cox proportional hazard regression models with age as the underlying time axis adjusted for sex, race/ethnicity, and clinic site. Full models were also adjusted for education, social activity, smoking status, body mass index, lipid-lowering medication use, history of cardiovascular disease, diabetes, Center for Epidemiologic Studies Depression Scale score, treatment group assignment, and *APOE* genotype.

Among participants with MCI at baseline, the HR for dementia was 1.72 (95% CI, 0.87-3.40) for more than 14.0 drinks per week compared with less than 1.0 drink per week. A formal test of linear trend of dementia risk according to numbers of alcoholic drinks per week was not significant (HR, 1.02; 95% CI, 0.99-1.05; *P* = .13). For 7.1 to 14.0 drinks per week vs less than 1.0 drink per week, the HR was 0.63 (95% CI, 0.38-1.06) among participants without MCI vs 0.93 (95% CI, 0.47-1.84) among participants with MCI. Accounting for the competing risk of death in the analysis did not alter the results (eTable 1 in the Supplement).

With regard to the risk of AD specifically, the patterns of association of reported alcohol consumption and risk for AD were similar to results for all-cause dementia, albeit with less precision. We also tested whether associations between alcohol consumption and all-cause dementia or AD risk differed by age, sex, or *APOE* genotype and found no evidence of effect size modification (all *P* for interaction >.05). Analyses stratified by *APOE* genotype are provided in eTable 2 in the Supplement. We found similar associations of alcohol use and risk of all-cause dementia or AD in analyses restricted to *APOE E4* noncarriers.

To explore the separate dimensions of quantity and frequency of alcohol consumption and their associations with dementia risk, participants who consumed a single beverage type were separated into categories of drinks per drinking day (Table 3 and eTable 3 and eTable 4 in the Supplement). We then contrasted the risk of dementia among those who consumed more than the recommended quantities per drinking day (ie, ≥2 drinks) on a less-than-daily basis with those consuming only 1 drink but daily (ie, potentially similar amounts overall volumes per week). Daily low-quantity drinking was associated with a lower dementia risk (HR, 0.45; 95% CI, 0.23-0.89; *P* = .02) compared with infrequent higher-quantity drinking.

**Table 3.** Adjusted HRs of All-Cause Dementia According to Self-reported Frequency of Alcohol Consumption and Quantity of Alcohol Consumed per Drinking Day in a Subset of 2095 Participants Without Cognitive Impairment at Baseline Who Consumed Preferentially 1 Beverage Type^a^

Characteristic	Drinking d/wk					
	Nondrinkers	<1	1-6		7	
			1 Drink/d	≥2 Drinks/d	1 Drink/d	≥2 Drinks/d
Participants, No.						
With dementia	151	36	24	14	23	28
Without dementia	875	238	191	71	217	227
HR (95% CI)	1.18 (0.81-1.72)	1 [Reference]	0.93 (0.55-1.57)	1.54 (0.82-2.90)	0.69 (0.40-1.19)	1.03 (0.61-1.71)

Abbreviation: HR, hazard ratio.

^a^ HRs were obtained from Cox proportional hazard regression models with age as the underlying time axis adjusted for sex, race/ethnicity, clinic site, education, social activity, smoking status, body mass index, lipid-lowering medication use, history of cardiovascular disease, diabetes, Center for Epidemiologic Studies Depression Scale score, treatment group assignment, and *APOE* genotype.

### Alcohol Consumption and Cognitive Decline

The association between alcohol intake and 3MSE scores was significantly modified by MCI at baseline (*P* for interaction = .02), but not by *APOE* genotype (*P* for interaction = .08). Among participants without MCI at baseline, nondrinkers had statistically significantly lower (worse) 3MSE scores during follow-up relative to baseline compared with participants consuming less than 1.0 drink per week (adjusted mean difference, −0.47 point; 95% CI, −0.88 to −0.05 point) (Table 4), but differences across other groups were inconsistent. Among participants with MCI at baseline, participants consuming more than 14.0 drinks per week had statistically significantly lower 3MSE scores compared with participants consuming less than 1.0 drink per week at follow-up compared with baseline (between-group difference, −3.49 points; 95% CI, −5.72 to −1.27 points). These differences persisted after adjustment for potential confounders. Compared with drinking less than 1.0 drink per week, complete abstention (in participants without MCI) and the consumption of more than 14.0 drinks per week (in participants with MCI) were associated with lower 3MSE scores (adjusted mean difference at follow-up compared with baseline, −0.46 point [95% CI, −0.87 to −0.04 point] and −3.51 points [95% CI, −5.75 to −1.27 points], respectively). The difference in 3MSE scores at follow-up compared with baseline by alcohol consumption was statistically significant only among participants with MCI at baseline (difference in scores, nondrinkers, −0.87 [95% CI, −0.29 to 0.56]; 1.0-7.0 drinks per week, −1.58 [95% CI, −3.28 to 0.11]; 7.1-14.0 drinks per week, −0.01 [95% CI, −2.15 to 2.13]; >14.0 drinks per week, −3.51 [95% CI, −5.75 to −1.27]; *P* for difference = .02) (Table 4). Scores for ADAS-Cog did not differ statistically significantly at follow-up compared with baseline by alcohol consumption in participants with MCI (difference in scores, nondrinkers, −0.12 [95% CI, −0.84 to 0.61]; 1.0-7.0 drinks per week, 0.05 [95% CI, −0.81 to 0.91]; 7.1-14.0 drinks per week, −0.18 [95% CI, −1.26 to 0.90]; >14.0 drinks per week, 0.48 [95% CI, −0.68 to 1.63]; *P* for difference = .82) or without MCI (difference in scores, 1.0-7.0 drinks per week, 0.06 [95% CI, −0.18 to 0.30]; 7.1-14.0 drinks per week, 0.06 [95% CI, −0.24 to 0.37]; >14.0 drinks per week, −0.10 [95% CI, −0.40 to 0.19]; *P *= .40) at baseline (Table 4). The association of alcohol intake and ADAS-Cog scores was not modified by MCI at baseline or *APOE* genotype (*P* for interaction = .58 and .26, respectively).

**Table 4.** Difference in Cognitive Scores at Follow-up According to Self-reported Usual Alcohol Consumption of 3006 Ginkgo Evaluation of Memory Study Participants

Characteristic	Difference (95% CI) in Cognitive Scores at Follow-up According to Weekly No. of Drinks					*P* Value for Difference
	Nondrinkers	0.1-0.9 Drinks/wk	1.0-7.0 Drinks/wk	7.1-14.0 Drinks/wk	>14.0 Drinks/wk	
No cognitive impairment at baseline						
Modified Mini-Mental State Examination score (n = 2509)						
Basic model^a^	−0.47 (−0.88 to −0.05)	0	−0.41 (−0.87 to 0.04)	0.12 (−0.45 to 0.69)	−0.23 (−0.78 to 0.33)	.06
Full model^b^	−0.46 (−0.87 to −0.04)	0	−0.41 (−0.86 to 0.05)	0.14 (−0.43 to 0.71)	−0.22 (−0.77 to 0.34)	.06
Alzheimer’s Disease Assessment Scale cognitive score (n = 2548)						
Basic model^a^	0.15 (−0.08 to 0.37)	0	0.07 (−0.18 to 0.31)	0.07 (−0.24 to 0.37)	−0.10 (−0.39 to 0.20)	.39
Full model^b^	0.14 (−0.08 to 0.36)	0	0.06 (−0.18 to 0.30)	0.06 (−0.24 to 0.37)	−0.10 (−0.40 to 0.19)	.40
Mild cognitive impairment at baseline						
Modified Mini-Mental State Examination score (n = 455)						
Basic model^a^	−0.87 (−2.29 to 0.55)	0	−1.59 (−3.28 to 0.09)	0.01 (−2.12 to 2.14)	−3.49 (−5.72 to −1.27)	.02
Full model^b^	−0.87 (−2.29 to 0.56)	0	−1.58 (−3.28 to 0.11)	−0.01 (−2.15 to 2.13)	−3.51 (−5.75 to −1.27)	.02
Alzheimer’s Disease Assessment Scale cognitive score (n = 473)						
Basic model^a^	−0.12 (−0.85 to 0.60)	0	0.06 (−0.80 to 0.92)	−0.16 (−1.24 to 0.92)	0.47 (−0.68 to 1.63)	.81
Full model^b^	−0.12 (−0.84 to 0.61)	0	0.05 (−0.81 to 0.91)	−0.18 (−1.26 to 0.90)	0.48 (−0.68 to 1.63)	.82

^a^ Difference in Modified Mini-Mental State Examination score or Alzheimer’s Disease Assessment Scale cognitive score at follow-up obtained from linear mixed models adjusted for age, sex, race/ethnicity, and clinic site.

^b^ Also adjusted for education, social activity, smoking status, body mass index, lipid-lowering medication use, history of cardiovascular disease, diabetes, depression (Center for Epidemiologic Studies Depression Scale score), treatment group assignment, and *APOE* genotype.

## Discussion

In this prospective cohort study of 3021 older adults, the association between self-reported alcohol consumption and dementia risk differed significantly depending on baseline cognitive status and amount of alcohol consumption. For 7.1 to 14.0 drinks per week compared with less than 1.0 drink per week, the HRs for dementia were 0.63 (95% CI, 0.38-1.06) among 2548 participants without MCI and 0.93 (95% CI, 0.47-1.84) among 473 participants with MCI. Among participants with MCI, the HR for dementia was 1.72 (95% CI, 0.87-3.40) for more than 14.0 drinks per week compared with less than 1.0 drink per week. In analyses of drinking patterns, daily low-quantity drinking was associated with lower dementia risk than infrequent higher-quantity drinking among participants without MCI at baseline.

A recent meta-analysis^3^ suggested a U-shaped pattern for the association between drinking and dementia, with a nadir at 4 drinks per week, whereas consuming 23 drinks per week or more was associated with a higher dementia risk, results generally consistent with our findings among participants without baseline MCI. At the same time, our results did not show significant associations and clearly do not suffice to suggest a clinical benefit from even limited alcohol use. Nonetheless, our findings provide some reassurance that alcohol consumed within recommended limits was not associated with an increased risk of dementia among older adults with normal baseline cognition. We were unable to assess the risks associated with excessive drinking. Over prolonged periods, excessive drinking may be associated with brain damage via direct and indirect effects of alcohol on the brain that may result in a range of conditions, including Wernicke-Korsakoff syndrome and alcoholic dementia.^25,26^ Because alcohol-associated brain damage is at least partially reversible when individuals maintain sobriety over extended periods,^27,28^ continued efforts to reduce excessive alcohol intake are essential, regardless of age.

The associations of self-reported alcohol consumption with dementia risk and cognitive decline were more consistently adverse among individuals with MCI than those with normal cognition. This was particularly true for the subset of individuals who drank more than 14.0 servings per week, whose rate of cognitive decline and risk of dementia were the highest of any subgroup. Of note, nearly one-half of participants with MCI reported current alcohol consumption, suggesting that it is not an infrequent behavior. Thus far, few studies have assessed alcohol consumption and dementia specifically among participants with MCI, and previous work has yielded inconsistent results. In the Italian Longitudinal Study on Aging,^10^ consumption of alcohol vs nonconsumption was associated with lower dementia risk among participants with MCI, but the analysis was based on only 14 patients with dementia. In a study^11^ of 176 male participants with MCI, no significant difference was found between abstainers, light-to-moderate drinkers, or excessive drinkers (> 12 drinks per week) and dementia risk over 2 years of follow-up. Light-to-moderate drinking compared with nondrinking has also been associated with an increased risk of conversion from MCI to AD.^12^ Given the significantly higher risk of cognitive decline among heavily drinking participants with MCI observed here, and the lack of a clear departure from linearity, we caution particular care for individuals with MCI who continue to drink alcohol.

Evidence linking drinking patterns to dementia risk has been sparse, although binge drinking has been associated with higher dementia risk.^5^ The findings of the current study, that infrequent higher-quantity drinking (≥2 drinks on drinking days) was associated with increased dementia risk, similar to binge drinking, highlight the risks of excessive drinking among older adults. Indeed, both the National Institute on Alcohol Abuse and Alcoholism^9^ and American Geriatrics Society^29^ suggest a limit of 7 drinks per week among older adults who consume alcohol. This finding also parallels the observation that low-quantity frequent drinking tends to be associated with the lowest cardiovascular disease and type 2 diabetes risk,^4,30^ which share several common risk factors with dementia.^31^

Several epidemiologic studies have assessed whether the association of alcohol intake with dementia risk and cognitive decline is modified by *APOE* genotype, but results were inconsistent.^32,33^ Some studies have found that moderate alcohol intake is associated with a lower risk of dementia or cognitive decline among *APOE E4* allele noncarriers and with a higher risk or absence of association among carriers.^6,34,35,36^ Some studies^37,38,39^ found no significant interaction between dementia and *APOE E4*, but a tendency for a lower dementia risk among *APOE E4* allele carriers has been noted elsewhere.^7^ A possible association might be more pronounced in younger populations, given that null findings, including those of our study, largely came from study populations aged 70 years and older^38,39^; further studies are needed to clarify the association.

A particular strength of GEMS is the systematic, prespecified, and comprehensive manner with which participants were followed for neurocognitive changes. As part of a clinical trial, we relied on a frequency and depth of cognitive assessment that few other studies can match. Other strengths include the low dropout and loss to follow-up rates (6.3%), as well as the comprehensive control for potential confounders, such as depressive symptoms and social interactions, and effect modifiers, including *APOE* genotype.

### Limitations

Our results should be viewed in the context of several limitations. The respective study population consists of participants near age 80 years who were all dementia free at study entry, thus limiting the generalizability to younger populations and carrying the potential for differential enrollment and survival. The risks associated with excessive drinking could not be assessed in the present study. Furthermore, we cannot exclude the possibility of residual confounding. Alcohol consumption was self-reported at the baseline examination only, and we could not assess changes over time. Studies investigating lifetime drinking habits following up participants into the oldest age range are warranted. Of importance, cognitive abilities might affect the validity of self-reported alcohol intake, although we observed the expected association of alcohol intake with high-density lipoprotein cholesterol values. Per study protocol, cognitive testing was discontinued after dementia diagnosis, restricting the analysis to cognitive decline before dementia diagnosis. Furthermore, we had no information on drinking history and we were not able to separate lifetime abstainers from recent ex-drinkers. The nondrinker category includes former drinkers and lifelong abstainers. Former drinkers may have given up drinking for health reasons not necessarily associated with their previous alcohol intake, but concern remains that they differ in systematic and difficult-to-control ways.^40^ Therefore, we used occasional drinkers as a referent as recommended by others. However, we cannot exclude that our results reflect an inverse association because health status—especially given the long preclinical phase of dementia—could determine drinking behavior. Specifically, we adjusted for history of lipid-lowering medication use, cardiovascular disease, diabetes, and depression but did not have a complete inventory of comorbidities. Thus, at present, our findings cannot be directly translated into clinical recommendations, and these findings warrant additional studies to confirm these associations further.

## Conclusions

In this study of older adults, the association of self-reported alcohol consumption with dementia risk appeared to cluster into 3 separate dimensions—baseline cognition, dose, and pattern. Among participants without MCI at baseline, daily low-quantity drinking was associated with lower dementia risk than infrequent higher-quantity drinking. Among participants with MCI, consumers of more than 14.0 drinks per week had the most severe cognitive decline compared with consumers of less than 1.0 drink per week. These results suggest that while caring for older adults, physicians should carefully assess the full dimensions of drinking behavior and cognition when providing guidance to patients about alcohol consumption.

## Notes

Supplement. **eAppendix 1.** Methods of Beverage Type Analysis **eAppendix 2.** Results of Beverage Type Analysis **eTable 1.** Adjusted Subhazard Ratios (sHR) and 95% CIs of Dementia According to Usual Alcohol Consumption of 3021 Participants of the Ginkgo Evaluation of Memory Study **eTable 2.** Hazard Ratios (HRs) and 95% CIs of Dementia According to Usual Alcohol Consumption and *APOE* Genotype in Participants of the Ginkgo Evaluation of Memory Study **eTable 3.** Adjusted Hazard Ratios (HRs) and 95% CIs of Alzheimer’s Disease According to Frequency of Alcohol Consumption and Quantity of Alcohol Consumed per Drinking Day in a Subset of Participants That Consumed Preferentially 1 Beverage Type **eTable 4.** Adjusted Hazard Ratios (HRs) and 95% CIs of Dementia According to Individual Alcoholic Beverages in 2548 Ginkgo Evaluation of Memory Study Participants Without Mild Cognitive Impairment at Baseline
