# Supplementary material for: Alcohol Consumption and Risk of Dementia and Cognitive Decline Among Older Adults With or Without Mild Cognitive Impairment
Source: JAMA Netw Open. 2019 Sep 27;2(9):e1910319. doi: 10.1001/jamanetworkopen.2019.10319 (PMC6777245; doi:10.1001/jamanetworkopen.2019.10319)
Supplement: Supplement. — eAppendix 1. Methods of Beverage Type Analysis eAppendix 2. Results of Beverage Type Analysis eTable 1. Adjusted Subhazard Ratios (sHR) and 95% CIs of Dementia According to Usual Alcohol Consumption of 3021 Participants of the Ginkgo Evaluation of Memory Study eTable 2. Hazard Ratios (HRs) and 95% CIs of Dementia According to Usual Alcohol Consumption and APOE Genotype in Participants of the Ginkgo Evaluation of Memory Study eTable 3. Adjusted Hazard Ratios (HRs) and 95% CIs of Alzheimer’s Disease According to Frequency of Alcohol Consumption and Quantity of Alcohol Consumed per Drinking Day in a Subset of Participants That Consumed Preferentially 1 Beverage Type eTable 4. Adjusted Hazard Ratios (HRs) and 95% CIs of Dementia According to Individual Alcoholic Beverages in 2548 Ginkgo Evaluation of Memory Study Participants Without Mild Cognitive Impairment at Baseline [file jamanetwopen-2-e1910319-s001.pdf]

Supplementary Online Content

Koch M, Fitzpatrick AL, Rapp SR, et al. Alcohol consumption and risk of dementia and cognitive decline among older adults with or without mild cognitive impairment. *JAMA Netw Open*. 2019;2(9):e1910319. doi:10.1001/jamanetworkopen.2019.10319

eAppendix 1. Methods of Beverage Type Analysis

eAppendix 2. Results of Beverage Type Analysis

eTable 1. Adjusted Subhazard Ratios (sHR) and 95% CIs of Dementia According to Usual Alcohol Consumption of 3021 Participants of the Ginkgo Evaluation of Memory Study

eTable 2. Hazard ratios (HRs) and 95% CIs of Dementia According to Usual Alcohol Consumption and *APOE* Genotype in Participants of the Ginkgo Evaluation of Memory Study

eTable 3. Adjusted Hazard Ratios (HRs) and 95% CIs of Alzheimer's Disease According to Frequency of Alcohol Consumption and Quantity of Alcohol Consumed per Drinking Day in a Subset of Participants That Consumed Preferentially 1 Beverage Type

eTable 4. Adjusted Hazard Ratios (HRs) and 95% CIs of Dementia According to Individual Alcoholic Beverages in 2548 Ginkgo Evaluation of Memory Study Participants Without Mild Cognitive Impairment at Baseline

This supplementary material has been provided by the authors to give readers additional information about their work.

© 2019 Koch M et al. *JAMA Network Open.*

eAppendix 1. Methods of Beverage Type Analysis

To determine whether alcoholic beverage types differ in their association with risk of all-cause dementia, and AD specifically, we categorized participants by the beverage that they consumed preferentially (≥ 80 % of total intake). If no alcoholic beverage type constituted 80% of the total intake, we classified participants as non-preferential drinkers. In a subset of participants that preferentially consumed one beverage type, we examined the frequency of consumption and quantity consumed per drinking day as separate exposures controlling for the intake of the other beverages; among non-preferential drinkers, we could not assess overall drinking frequency and quantity due to the exclusive collection of beverage-specific frequencies and quantities.

eAppendix 2. Results of Beverage Type Analysis

The risk of AD did not differ significantly by drinking quantity per drinking day (eTable 2; p for difference = 0.76). We tested if the association of alcohol consumption and dementia differed by beverage type (eResults; eTable 3). Tests for differences in the risk for all-cause dementia or AD comparing the highest intake categories of the three beverage types were not statistically significant (p for difference = 0.21 and 0.31).

© 2019 Koch M et al. *JAMA Network Open.*

eTable 1. Adjusted Subhazard Ratios (sHR)[a] and 95% CIs of Dementia According to Usual Alcohol Consumption in 3021 Participants of the Ginkgo Evaluation of Memory Study.

	Weekly Number of Drinks					P-value for trend	
	None	0.1-0.9	1.0-7.0	7.1-14.0	>14	Linear	Quadratic
**Dementia**							
No cognitive impairment at baseline, N=2548							
Basic model, sHR (95%CI)	1.12 (0.82, 1.53)	1.00	0.91 (0.63, 1.30)	0.66 (0.40, 1.08)	0.81 (0.51, 1.30)	0.07	0.21
Full model, sHR (95%CI)	1.12 (0.82, 1.55)	1.00	0.84 (0.58, 1.22)	0.62 (0.37, 1.04)	0.82 (0.51, 1.32)	0.07	0.11
MCI at baseline, N=473							
Basic model, sHR (95%CI)	1.00 (0.65, 1.56)	1.00	1.02 (0.61, 1.71)	1.21 (0.61, 2.41)	1.69 (0.91, 3.11)	0.07	0.99
Full model, sHR (95%CI)	0.93 (0.60, 1.45)	1.00	0.93 (0.55, 1.55)	0.99 (0.48, 2.07)	1.73 (0.91, 3.27)	0.09	0.63

[a]Subhazard Ratios obtained from Fine and Gray proportional subhazard regression models (death used as a competing risk) with age as the underlying time axis adjusted for sex, race/ethnicity, and clinic site. Full models were additionally adjusted for education, social activity, smoking status, body mass index, lipid-lowering medication use, history of cardiovascular disease, diabetes, Center for Epidemiologic Studies-Depression Scale, treatment arm assignment, and *APOE* genotype.
Abbreviations: MCI, Mild cognitive impairment.

© 2019 Koch M et al. *JAMA Network Open.*

eTable 2: Hazard Ratios (HRs)[a] and 95% CIs of Dementia According to Usual Alcohol Consumption and *APOE* Genotype in Participants of the Ginkgo Evaluation of Memory Study

		Weekly Number of Drinks				
	N	None	0.1-0.9	1.0-7.0	7.1-14.0	>14
APOE E4 allele carrier, HR (95%CI)	571	0.93 (0.57, 1.50)	1.00	0.74 (0.43, 1.26)	0.77 (0.37, 1.61)	0.77 (0.34, 1.76)
APOE E4 allele non-carrier, HR (95%CI)	1845	1.27 (0.85, 1.90)	1.00	1.05 (0.66, 1.67)	0.93 (0.52, 1.69)	1.45 (0.86, 2.46)

[1]Hazard Ratio obtained from Cox proportional hazard regression models with age as the underlying time axis or linear mixed models adjusted for sex, race/ethnicity, clinic site, education, social activity, smoking status, body mass index, lipid-lowering medication use, history of cardiovascular disease, diabetes, Center for Epidemiologic Studies-Depression Scale, and treatment arm assignment. Participants with missing APOE genotype information were excluded.

© 2019 Koch M et al. *JAMA Network Open.*

eTable 3. Adjusted Hazard Ratios (HRs)[a] and 95% CIs of Alzheimer's Disease According to Frequency of Alcohol Consumption and Quantity of Alcohol Consumed per Drinking Day in a Subset of Participants That Consumed Preferentially 1 Beverage Type (N=2509)

	**Drinking days/week**								
	**None**		**<1**		**1-6**			**7**	
					1 drink/ drinking day	≥ 2 drinks/ drinking day		1 drink/ drinking day	≥ 2 drinks/ drinking day
N participants with Alzheimer's disease	169		38		26	12		33	29
HR (95%CI)	1.14 (0.79, 1.63)		1.00		0.95 (0.57, 1.58)	1.04 (0.53, 2.02)		0.93 (0.58, 1.51)	1.02 (0.62, 1.69)

[1]Hazard Ratio obtained from Cox proportional hazard regression models with age as the underlying time axis adjusted for sex, race/ethnicity, clinic site, education, social activity, smoking status, body mass index, lipid-lowering medication use, history of cardiovascular disease, diabetes, Center for Epidemiologic Studies-Depression Scale, treatment arm assignment, and APOE genotype.

.

© 2019 Koch M et al. *JAMA Network Open.*

eTable 4. Adjusted Hazard Ratios (HRs)[a] and 95% CIs of Dementia According to Individual Alcoholic Beverages in 2548 Ginkgo Evaluation of Memory Study Participants Without Mild Cognitive Impairment at Baseline

		Weekly Number of Drinks					P-value for trend	
Type of Alcoholic Beverage		None	0.1-0.9	1.0-7.0	≥7.1		Linear	Quadratic
Dementia								
	Wine, HR (95%CI)	1.46 (1.03, 2.06)	1.00	1.02 (0.70, 1.49)	1.71 (0.87, 3.35)		0.55	0.03
	Beer, HR (95%CI)	0.96 (0.66, 1.40)	1.00	0.89 (0.57, 1.40)			0.18	0.60
	Liquor, HR (95%CI)	1.00 (0.68, 1.47)	1.00	0.85 (0.56, 1.29)			0.33	0.21
Alzheimer's disease								
	Wine, HR (95%CI)	1.24 (0.82, 1.89)	1.00	1.08 (0.68, 1.70)	2.26 (1.09, 4.71)		0.41	0.05
	Beer, HR (95%CI)	1.10 (0.69, 1.77)	1.00	1.13 (0.65, 1. 98)			0.16	0.04
	Liquor, HR (95%CI)	1.31 (0.78, 2.18)	1.00	1.23 (0.71, 2.11)			0.93	0.58

[1]Hazard Ratio obtained from Cox proportional hazard regression models with age used as the underlying time axis adjusted for sex, clinic site, race/ethnicity, education, social activity, smoking status, body mass index, lipid lowering medication use, history of cardiovascular disease, diabetes, depression (Center for Epidemiologic Studies-Depression Scale), and treatment arm assignment, and *APOE* genotype.

© 2019 Koch M et al. *JAMA Network Open.*
